# Correction: THRUST: translesion synthesis-driven hierarchical regulation using a template-activator construct for Cas12a activity

**DOI:** 10.1039/d5sc90280k

**Published:** 2026-01-09

**Authors:** Lulu Qin, Wen-Jin Wang, Xinyi Xia, Tongshan Zuo, Yilin Cai, Guanhong Xu, Fangdi Wei, Suling Wang, Qin Hu, Zheng Zhao, Fan Zhang, Ben Zhong Tang, Yao Cen

**Affiliations:** a School of Pharmacy, Nanjing Medical University Nanjing Jiangsu 211166 China yaocen@njmu.edu.cn; b Clinical Translational Research Center of Aggregation-Induced Emission, School of Medicine, The Second Affiliated Hospital, School of Science and Engineering, Shenzhen Institute of Aggregate Science and Technology, The Chinese University of Hong Kong, Shenzhen (CUHK-Shenzhen) Guangdong 518172 China tangbenz@cuhk.edu.cn; c Clinical Medical Laboratory Center, Department of Neurology, The Affiliated Taizhou People's Hospital of Nanjing Medical University, Taizhou School of Clinical Medicine, Nanjing Medical University Taizhou Jiangsu 225300 China; d State Key Laboratory of Natural Medicines, Jiangsu Provincial Key Laboratory for TCM Evaluation and Translational Research, School of Traditional Chinese Pharmacy, China Pharmaceutical University Nanjing Jiangsu 211198 China zhangfan@cpu.edu.cn

## Abstract

Correction for ‘THRUST: translesion synthesis-driven hierarchical regulation using a template-activator construct for Cas12a activity’ by Lulu Qin *et al.*, *Chem. Sci.*, 2025, **16**, 12917–12926, https://doi.org/10.1039/D5SC02575C.

The authors regret that the email addresses of the corresponding authors were not included in their original publication. The corresponding authors' email addresses are now provided in the corrected ‘Author affiliations’ herein and are also copied below:

Yao Cen – yaocen@njmu.edu.cn

Ben Zhong Tang – tangbenz@cuhk.edu.cn

Fan Zhang – zhangfan@cpu.edu.cn

The Royal Society of Chemistry apologises for these errors and any consequent inconvenience to authors and readers.

